# Supplementary material for: COVID-19 vaccine prioritization of incarcerated people relative to other vulnerable groups: An analysis of state plans
Source: PLoS One. 2021 Jun 15;16(6):e0253208. doi: 10.1371/journal.pone.0253208 (PMC8205184; doi:10.1371/journal.pone.0253208)
Supplement: S1 Table — (DOCX) [file pone.0253208.s001.docx]

**S3 Table**. **Coding of state’s relative prioritization of people who are incarcerated, correctional staff, law enforcement, people ≥65, and LTCF residents**.

| **State** | **Correctional facilities/departments included as key partners for reaching critical populations?** | **Phase of Priority** | | | | |
| --- | --- | --- | --- | --- | --- | --- |
|  |  | **People who are incarcerated** | **Correctional staff** | **Law enforcement** | **People age >65** | **LTCF** |
| **Alabama** | Yes | 1B | 1B | 1B | 1C^a^ | 1A |
| **Alaska** | NS | NS | NS | NS | NS | 1A |
| **Arizona** | Yes | NS | 1B | 1B | 1C | 1A |
| **Arkansas** | Yes | NS | 1B | NS | 1C | 1A |
| **California** | Yes | NS | NS | NS | 1B | 1A |
| **Colorado** | Yes | NS | 1B | 1B | 2 | 1A |
| **Connecticut** | Yes | NS | NS | 1A^b^ | NS | 1A |
| **Delaware** | Yes | 1C | 1C | NS | NS | 1A |
| **Florida** | Yes | NS | NS | 1 | NS | 1 |
| **Georgia** | Yes | 2 | 2 | 1B | 1C | 1A |
| **Hawaii** | Yes | 2 | 2 | 1A | 2 | 1B^c^ |
| **Idaho** | Yes | 2 | 1B | 1B | NS | 1A |
| **Illinois** | Yes | NS | 1B | 1B | 1C | 1A |
| **Indiana** | Yes | 2 | 2 | 2 | 1B | 1B |
| **Iowa** | Yes | 2 | 2 | 1B | 1B | 1A |
| **Kansas** | Yes | NS | NS | NS | 1B | 1B |
| **Kentucky** | Yes | 1B | 1B | NS | 1C^d^ | 1A |
| **Louisiana** | Yes | 2 | 1B | 1B | NS | 1B |
| **Maine** | Yes | 2 | 2 | NS | 1C^e^ | 1A |
| **Maryland** | NS | 1 | 1 | 1 | 2^f^ | 1 |
| **Massachusetts** | Yes | 1 | 1 | 1 | 2 | 1 |
| **Michigan** | Yes | NS | 1B | 1B^g^ | 1C^h^ | 1A |
| **Minnesota** | NS | NS | NS | NS | NS | 1A |
| **Mississippi** | Yes | 2 | 2 | 1B^i^ | 2^j^ | 1B |
| **Missouri** | Yes | NS | 1B | 1B | 1B | 1A |
| **Montana** | Yes | 1B | NS | 1C | 1C^k^ | 1A |
| **Nebraska** | Yes | NS | NS | NS | 1C^l^ | 1A |
| **Nevada** | Yes | 1B | 1A | 1A | 1C | 1A |
| **New Hampshire** | Yes | NS | NS | 1A | NS | 1A |
| **New Jersey** | Yes | 1A | 1A | 1B | 1C^m^ | 1A |
| **New Mexico** | Yes | 1 | 1B | NS | 1A | 1B |
| **New York** | Yes | NS | NS | 2 | 3 | 1 |
| **North Carolina** | Yes | 2^n^ | 1B | 2^o^ | 2 | 1A |
| **North Dakota** | Yes | 2^p^ | 2^q^ | NS | 1C | 1A |
| **Ohio** | Yes | 2 | 2 | NS | 1B | 1A^r^ |
| **Oklahoma** | Yes | 2 | 2 | 2 | 2 | 1 |
| **Oregon** | Yes | NS | 1A | 1A | NS | 1A |
| **Pennsylvania** | NS | 1B | 1B | 1B | 1B | 1A |
| **Rhode Island** | Yes | 2^s^ | 1 | 1 | 2^t^ | 1 |
| **South Carolina** | Yes | NS | NS | 1A | 1B | 1A |
| **South Dakota** | Yes | NS | 1C | 1C | 1D | 1B |
| **Tennessee** | Yes | 3 | 2A | NS | 1B^u^ | 1A |
| **Texas** | Yes | NS | NS | NS | 1B | 1A |
| **Utah** | Yes | 2 | 2 | 1B | 2 | 1B |
| **Vermont** | Yes | NS | 1B | 1B | 1C | 1A |
| **Virginia** | Yes | NS | NS | 1B^v^ | 1B | 1A |
| **Washington** | Yes | 2 | 2 | 1 | 2^w^ | 1A^x^ |
| **Washington DC** | Yes | 1B | 1B | 1B | 1B | 1B |
| **West Virginia** | Yes | NS | 1B | 1B | 2A^y^ | 1A |
| **Wisconsin** | Yes | NS | NS | NS | 1B | 1B^z^ |
| **Wyoming** | Yes | NS | 1B^aa^ | 1B^bb^ | 1B^cc^ | 1A |

LTCF = Long Term Care Facility

NS = Not specified

1. People aged 75+ prioritized as phase 1B and people ages 65-74+ as phase 1C
2. Eligible “if actively responding to medical 911 calls or involved in care for COVID or suspected COVID cases.”
3. "Adults aged 65 and older living in congregate or overcrowded settings Centers for Medicaid and Medicare (CMS) HDOH (Office of Healthcare Assurance) HAH (Long‐term care)"
4. People aged 75+ prioritized as phase 1B and people aged 65-74+ years prioritized as phase 1C
5. People aged 75+ prioritized as phase 1B and people aged 65-74+ years prioritized as phase 1C
6. Prioritized for phase 1 and phase 2 in two different places in the plan.
7. Supplement prioritizes for phase 1B “First responders not covered in Phase 1A (e.g., firefighters, police)”
8. People aged 75+ prioritized as phase 1B and people aged 65-74+ years prioritized as phase 1C
9. Law enforcement prioritized for phase 1A and 1B at two different places in the plan.
10. Prioritized for phase 1B and phase 2 at two different places.
11. People aged 75+ prioritized as phase 1B and people aged 65-74+ years prioritized as phase 1C
12. People aged 75+ prioritized as phase 1B and people aged 65-74+ years prioritized as phase 1C
13. Prioritized for phase 1B and 1C in two different locations within the state plan.
14. Prioritization depends on health status/age. Phase 1B: Incarcerated individuals with 2+ Chronic Conditions* or > age 65. Phase 2: Incarcerated individuals without 2+ Chronic Conditions
15. Frontline workers with 2+ Chronic Conditions at high risk of exposure (including police) for phase 1B, Frontline workers at high or moderate risk of exposure without 2+ Chronic Conditions for phase 2
16. Plan states: “Corrections and homeless may be included in phase 1B or in phase 2.” Also states: Other congregate settings (i.e., corrections, shelters) will likely be vaccinated during phase 1C.”
17. Plan states: “Corrections and homeless may be included in phase 1B or in phase 2.” Also states: “Other congregate settings (i.e., corrections, shelters) will likely be vaccinated during phase 1C.”
18. Plan states “Residents and staff at nursing homes; residents and staff at assisted living facilities, patients and staff at state psychiatric hospitals”
19. Supplement prioritizes for phase 1 people who are incarcerated who are >65, immunocompromised, or have other risk factors
20. Plan prioritizes “all other older adults” for phase 2; supplement prioritizes adults ≥75 for phase 1.
21. Simultaneous age-based criteria include those 65+ beginning in phase 1B, and those 75+ beginning between phase 1A and 1B
22. Prioritizes for 1B: “Other law enforcement personnel not included in phase 1A”
23. Prioritized people ≥65 years old for phase 1B and older adults for phase 2
24. Prioritized for this phase: “Residents and staff of nursing homes, assisted living facilities, and other community-based, congregate living settings where most individuals over 65 years of age are receiving care, supervision, or assistance aiming to avoid hospitalizations, severe morbidity, and mortality.”
25. Prioritized those 80+, 70+, and 60+ within phase 2A.
26. Plan mentions some long term care facilities that do not have vaccinating capacity may receive the vaccine during phase 1A; otherwise prioritized for 1B
27. Prioritized for phase 1A, but 1B includes “Fire, police, 911, correctional staff, search and rescue, and other in-person emergency response personnel not included in Phase 1A”
28. Prioritized for phase 1A, but 1B includes “Fire, police, 911, correctional staff, search and rescue, and other in-person emergency response personnel not included in Phase 1A”
29. Prioritized “People who are >70 years of age or older. If necessary, populations may be broken down to vaccinate those who are >80 first”
