# Supplementary material for: COVID-19 vaccine prioritization of incarcerated people relative to other vulnerable groups: An analysis of state plans
Source: PLoS One. 2021 Jun 15;16(6):e0253208. doi: 10.1371/journal.pone.0253208 (PMC8205184; doi:10.1371/journal.pone.0253208)
Supplement: S1 Appendix — (DOCX) [file pone.0253208.s002.docx]

**S1 Appendix. List of state plans and supplementary documents analyzed.**

*PDFs of documents are available at: https://github.com/rachelstrodel/State-COVID-19-Vaccination-Plans*

| **STATE** | **MOST RECENT PLAN PUBLICATION (AS OF 12/31/20)** | **SUPPLEMENTARY DOCUMENTATION (DATE OF PUBLICATION)** |
| --- | --- | --- |
| Alabama | 11/06/2020 | Alabama COVID‐19 Vaccination Allocation Plan (12/23/2020) |
| Alaska | 10/16/2020 | Alaska Vaccine Allocation Committee Meeting Summary (12/03/2020) |
| Arizona | 12/14/2020 | Arizona COVID-19 Vaccine Distribution slides (No date) |
| Arkansas | 10/16/2020 | Arkansas Department of Health COVID-19 Vaccination Phased Plan (12/15/2020) |
| California | 10/16/2020 | Guidelines to Californias Health Departments Allocation of COVID-19 Vaccine During Phase 1A (12/14/2020) |
| Colorado | 10/16/2020 | Guidance on State COVID-19 Website (Checked 12/31/20) |
| Connecticut | 10/15/2020 | Guidance on State COVID-19 Website (Checked 12/31/20) |
| Delaware | 12/09/2020 | NA |
| District of Columbia | 11/27/2020 | NA |
| Florida | 10/16/2020 | Press Release: Governor Ron DeSantis Provides Update on COVID-19 Vaccine Distribution Plan (12/10/2020) |
| Georgia | 12/07/2020 | NA |
| Hawaii | 10/16/2020 | NA |
| Idaho | 10/19/2020 | Vaccine Advisory Committee Prioritization slides (12/18/2020) |
| Illinois | 12/04/2020 | NA |
| Indiana | 10/2020 | NA |
| Iowa | 12/04/2020 | NA |
| Kansas | 11/04/2020 | NA |
| Kentucky | 10/2020 | Vaccine Phase 1B Guidance Update (12/28/2020) |
| Louisiana | 10/16/2020 | NA |
| Maine | 10/16/2020 | Guidance on State COVID-19 Website (Checked 12/31/20) |
| Maryland | 10/16/2020 | NA |
| Massachusetts | 10/16/2020 | Supplementary Presentation Slides (12/09/2020) |
| Michigan | 10/16/2020 | Michigan COVID-19 Vaccination Interim Prioritization Guidance (12/23/2020) |
| Minnesota* | 10/21/20 | Supplementary Phase 1A Guidance (12/08/2020) |
| Mississippi | 10/16/2020 | NA |
| Missouri | 11/11/2020 | NA |
| Montana | 10/16/2020 | Proposed COVID-19 Vaccine Allocation – Montana (12/24/20) |
| Nebraska | 12/31/2020 | NA |
| Nevada | 12/01/2020 | NA |
| New Hampshire | 10/30/2020 | Supplementary Phase 1 Guidance (12/24/2020) |
| New Jersey | 12/15/2020 | Priority Group Supplement (Date Unknown) |
| New Mexico | 10/16/2020 | NA |
| New York | 10/2020 | NA |
| North Carolina | 10/16/2020 | Supplementary Phase 1A Guidance (12/22/2020) |
| North Dakota | 12/11/2020 | Supplementary Phase 1A Guidance (12/16/2020) |
| Ohio | 10/16/2020 | Supplementary Phase 1A Guidance (Date Unknown) Supplementary Phase 1B Guidance (Date Unknown) |
| Oklahoma | 10/14/2020 | COVID-19 Vaccine Priority Population Framework for Oklahoma (Date Unknown) |
| Oregon | 11/6/2020 | Supplementary Phase 1A Vaccine Sequencing Plan (12/18/2020) |
| Pennsylvania | 12/11/2020 | NA |
| Rhode Island | 10/16/2020 | WHO can get vaccinated in PHASE 1 of Rhode Island’s COVID-19 Vaccination Program? (Date unknown) |
| South Carolina | 12/07/2020 | Phase 1A Guidance for COVID-19 Vaccine Allocation (December 2020) |
| South Dakota | 12/14/2020 | COVID-19 Vaccine Priority Groups for Phase 1 (12/14/20) |
| Tennessee | 12/30/2020 | Guidance on State COVID-19 Website (Checked 12/31/20) |
| Texas | 10/16/2020 | COVID-19 Vaccine Allocation Phase 1A Definition (12/17/20)  COVID-19 Vaccine Allocation Phase 1B Definition (12/22/20) |
| Utah | 11/18/2020 | NA |
| Vermont | 12/28/2020 | NA |
| Virginia | 11/16/2020 | Virginia COVID-19 Vaccination Prioritization Guidance (12/04/2020) |
| Washington** | 10/20/2020 | WA State COVID-19 Vaccine Prioritization Guidance and Interim Allocation Framework (12/30/20) |
| West Virginia | 10/16/2020 | Distribution Timeline Slides (12/17/20) |
| Wisconsin | 10/2020 | NA |
| Wyoming | 11/25/2020 | Supplementary Phase 1A and 1B Prioritization Guidance (12/30/2020) |

*Only an Executive Summary of the State Plan was available.

**01/05/21 version included in database because 12/30/20 version was unavailable at time of download.
