# Supplementary material for: COVID-19 vaccine prioritization of incarcerated people relative to other vulnerable groups: An analysis of state plans
Source: PLoS One. 2021 Jun 15;16(6):e0253208. doi: 10.1371/journal.pone.0253208 (PMC8205184; doi:10.1371/journal.pone.0253208)
Supplement: S2 Appendix — (DOCX) [file pone.0253208.s003.docx]

**S2 Appendix. Code development and coding assumptions.**

1. *A priori codes and original questions from CDC Playbook*

We developed a priori codes based on the U.S. Centers for Disease Control and Prevention Vaccination Program Interim Playbook for Jurisdiction Operations. Relevant questions and sections of the Playbook guiding code development are listed below.

| **Section 2: COVID-19 Organizational Structure and Partner Involvement** | **Relevant a priori code** |
| --- | --- |
| C. Describe how your jurisdiction will plan for, develop, and assemble a broader committee of key internal leaders and external partners to assist with implementing the program, reaching critical populations, and developing crisis and risk communication messaging. | Are correctional facilities/departments included as key partners for reaching critical populations? |
| D. Identify and list members and relevant expertise of the internal team and the internal/external committee. |  |
| G. List key partners for critical populations that you plan to engage and briefly describe how you plan to engage them, including but not limited to:  • Pharmacies  • Correctional facilities/vendors  • Homeless shelters  • Community-based organizations |  |

| **Section 3: Phased Approach to COVID-19 Vaccination** | **Relevant a priori code** |
| --- | --- |
| Describe how your jurisdiction will structure the COVID-19 Vaccination Program around the three phases of vaccine administration: • Phase 1: Potentially Limited Doses Available • Phase 2: Large Number of Doses Available, Supply Likely to Meet Demand • Phase 3: Likely Sufficient Supply, Slowing Demand | At which phase are the following groups prioritized for receiving a COVID-19 vaccine?   - People who are incarcerated - Correctional staff - Law enforcement - People ≥65 years old - Long-term care facility residents |

| **Section 4: Critical Populations** | **Relevant a priori code** |
| --- | --- |
| A. Describe how your jurisdiction plans to: 1) identify, 2) estimate numbers of, and 3) locate (e.g., via mapping) critical populations. Critical population groups may include: • Healthcare personnel • Other essential workers • Long-term care facility residents (e.g., nursing home and assisted living facility residents) • People with underlying medical conditions that are risk factors for severe COVID-19 illness • People 65 years of age and older • People from racial and ethnic minority groups • People from tribal communities • People who are incarcerated/detained in correctional facilities • People experiencing homelessness/living in shelters • People attending colleges/universities • People living and working in other congregate settings • People living in rural communities • People with disabilities • People who are under- or uninsured | At which phase are the following groups prioritized for receiving a COVID-19 vaccine?   - People who are incarcerated - Correctional staff - Law enforcement - People ≥65 years old - Long-term care facility residents |
| B. Describe how your jurisdiction will define and estimate numbers of persons in the critical infrastructure workforce, which will vary by jurisdiction. |  |
| C. Describe how your jurisdiction will determine additional subset groups of critical populations if there is insufficient vaccine supply. |  |
| D. Describe how your jurisdiction will establish points of contact (POCs) and communication methods for organizations, employers, or communities (as appropriate) within the critical population groups. |  |

1. *Coding Assumptions*

Throughout the coding process, we resolved discrepancies and converged on a common set of assumptions to enhance interrater reliability. These assumptions are as follows:

- If ‘first responders,’ ‘essential workers,’ ‘critical infrastructure workforce’ were prioritized, they were not coded as law enforcement or correctional officers unless those groups were explicitly mentioned.
- If ‘public safety’ was a prioritized group, this was coded as law enforcement, but not correctional officers (e.g. Montana)
- If ‘critical populations’ or ‘people in congregate settings’ were explicitly included in state prioritization plans but not people who are incarcerated, in correctional settings, or in jail/prison, this was coded as ‘not specified.’
- If ‘Corrections’ was prioritized without distinguishing between correctional staff and people who are incarcerated, this was coded as being the phase of priority for both groups.
- If ‘older adults’ were mentioned without a specific age range, they were coded as the 65+ category.
- If the plans prioritized a certain group for two different phases within the same document, we coded them as being prioritized for the later of the two phases.
- If only a subset of a group was prioritized (e.g. “adults ≥65 years old with one or more chronic conditions”) this was not considered representative of the whole group.
- If age cutoffs were close to but did not exactly align with our coding schema, we included a footnote noting this in supplement 3. For age criteria, for example, if plans prioritized people 65-74 later than from people ≥75+, we included the 65-74yo phase as the phase of priority and included a footnote indicating this.
- If a state plan copied and pasted the prioritization tables/graphs from the ACIP or NASEM reports, they were only included in our analysis if the plan clearly stated they planned to use these frameworks and were not simply including them as examples of potential prioritization schemes.
- If a state added supplemental materials to its prioritization plan, we updated all fields to reflect the most recent priority guidance from that supplement.
