# Supplementary material for: COVID-19 vaccine prioritization of incarcerated people relative to other vulnerable groups: An analysis of state plans
Source: PLoS One. 2021 Jun 15;16(6):e0253208. doi: 10.1371/journal.pone.0253208 (PMC8205184; doi:10.1371/journal.pone.0253208)
Supplement: Attachment — Submitted filename: LeMasters_PLOS_One_Review.docx [file pone.0253208.s004.docx]

This is an important contribution to the literature on COVID-19 in carceral settings and the lack of prioritization of this vulnerable population.

Abstract:

- Do you have space in the abstract to also explicitly say the number of states that have placed incarcerated populations in group 3 priority?

Introduction:

- Limited vaccine availability is mentioned as a motivation. Given that the number of vaccines available has greatly increased in the past few months, I’m wondering if you could include something about how this is even a more pressing now that vaccines are not in as short of supply and incarcerated populations remain under-prioritized.
- The interpretation of case rates should be 5.5 times *that of* vs. 5.5 times *higher* (line 56).
- When discussing community spread in the introduction, could you add that communities that incarcerated individuals go home to bear a disproportionate burden of COVID-19? This brings together the need to both decarcerate and prioritize vaccination – they cannot happen separately.
- You discuss workforce exposure but neglect to mention that incarcerated persons have worked throughout the pandemic – sometimes in jobs that in direct contact with COVID-19 (e.g., burial services). It would be helpful to include this piece as well.

Methods:

- While listed in the limitations section, I would add in the methods section that it is unclear if this analysis captures all incarcerated persons or only a subset. Given that it is state wide guidance, I would think that this most likely captures those in state prisons rather than county jails, federal prisons, juvenile detention, etc.

Results:

- While the results are informative, I would be interested to see a sort of cross-tab. For instance, in states where prison and jail employees are prioritized, are those states also prioritizing incarcerated individuals? How much do these metrics track together – as this has clear implications for COVID-19 spread within these facilities.
- Similarly, it seems that, substantively, prioritizing 65+ and long-term facility care residents is slightly different than states prioritizing prison and jail employees and law enforcement, as the latter group’s prioritization directly affects COVID-19 spread in carceral settings. I would recommend framing the findings in this way.
- Please list data sources in appendix (e.g., website links).

Discussion:

- There is no mention of vaccine hesitancy among staff and incarcerated individuals, which has recently come to the forefront of many discussions (e.g., incarcerated persons with allergies may not trust the medical providers administering the vaccines to quickly respond to adverse effects; many correctional staff say that they will not take the vaccine; incarcerated persons have historically been used to test medicines and vaccines and rightly mistrust medical and public health systems). This seems important to discuss.
